# Comparing cryo-EM structures of the vertebrate cardiac muscle thick filament

**DOI:** 10.1007/s12551-025-01393-9

**Published:** 2026-01-08

**Authors:** Roger Craig, Debabrata Dutta, Natalia A. Koubassova, Andrey K. Tsaturyan, Raúl Padrón

**Affiliations:** 1 University of Massachusetts Chan Medical School, Worcester, MA USA; 2 Lomonosov Moscow State University, Moscow, Russia; 3 Tel Aviv University, Tel Aviv, Israel; 4 Imperial College London, London, UK

**Keywords:** Thick filament, Cryo-EM, Cryo-ET, Muscle, Myosin

## Abstract

The thick filaments of muscle carry the myosin motors that generate contraction by interacting with actin filaments. Despite decades of research, an atomic model of the vertebrate striated muscle thick filament has been lacking. Three cryo-EM thick filament structures from cardiac muscle have now been reported, revealing the organization of myosin heads in interacting-heads motifs (IHMs) on the thick filament surface, and a complex arrangement of myosin tails, titin, and myosin-binding protein C (MyBP-C) in the filament backbone. The three studies are complementary, coming from two species (mouse and human), two techniques (cryo-electron tomography and single particle cryo-EM), and from muscles treated or untreated with the myosin-stabilizing drug mavacamten. The structures are remarkably similar, agreeing on most elements of molecular organization but differing on the degree of stability of the IHMs and the organization of MyBP-C. The differences arise from the different techniques used and the observation of filaments within, or isolated from, the filament lattice.

**Supplementary Information:**

The online version contains supplementary material available at 10.1007/s12551-025-01393-9.

## Introduction

**Fig. 1:**
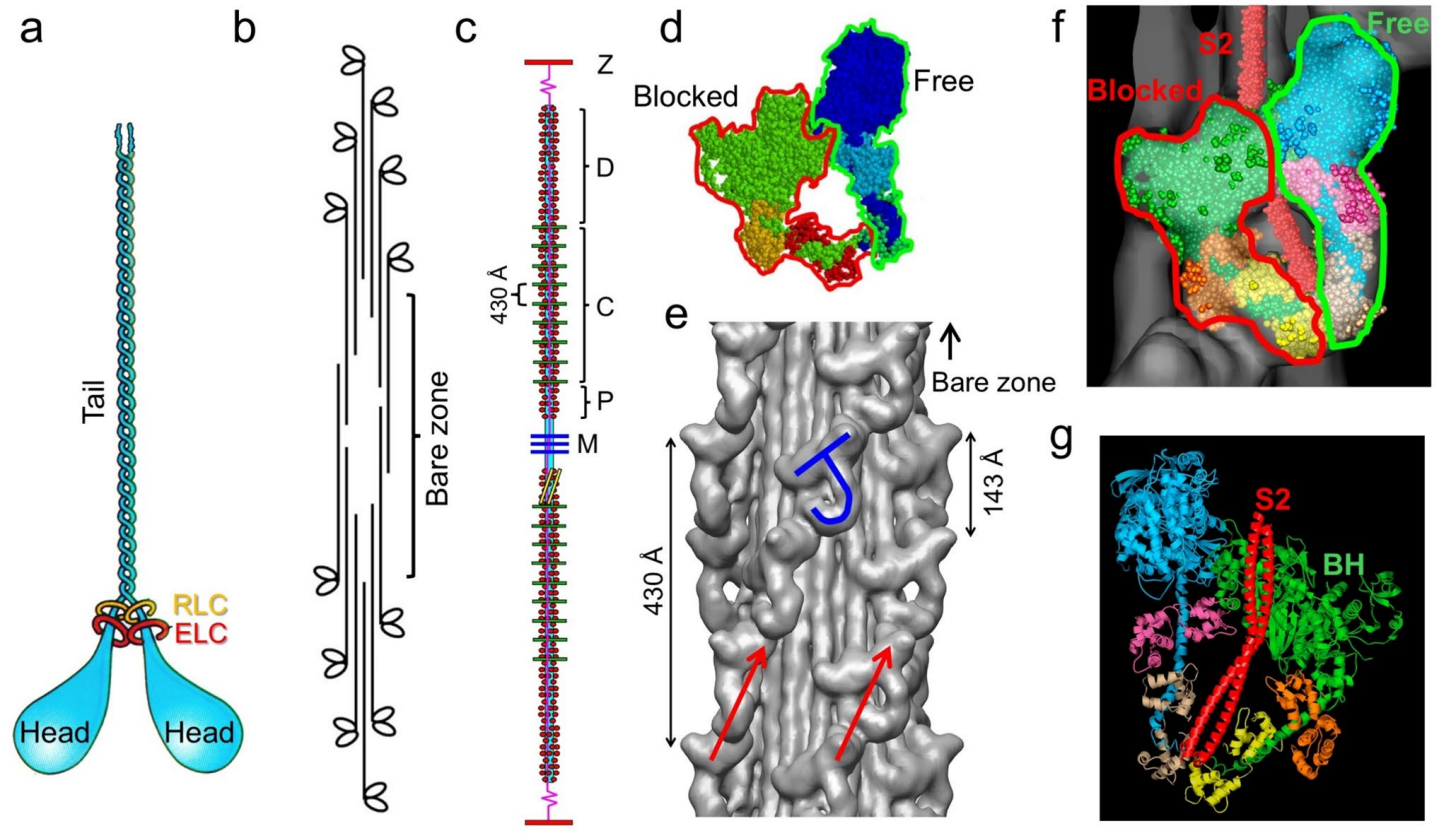
Myosin and thick filament structure. (**a**) Myosin 2 molecule showing tail, heads, and light chains. (**b**) Basic structure of bipolar thick filament, with tails in backbone, heads on surface, and central bare zone. (**c**) Vertebrate filament showing ordering of heads with a 430 Å repeat, and associated proteins MyBP-C, defining the C-zone (C), and titin. Red spheres represent pairs of myosin heads in quasi-helices (yellow lines), green bars show 430-Å spaced positions of MyBP-C, backbone is cyan, titin (pink) runs along each half thick filament and has spring-like properties beyond the filament tip, in the I-band. Z-line, M-line, P zone and D zone are also marked. (**d**) PDB 1i84: Interacting heads (blocked and free), first identified by cryo-EM of 2D crystals of smooth muscle heavy meromyosin (Wendt et al. 2001). BH and FH heavy chains are green and dark blue; light chains are in narrow neck region of each head. S2 has been omitted from the PDB as its position was unknown. (**e**) Cryo-EM reconstruction of tarantula thick filament (Woodhead et al. 2005) showing repeating J-shaped motif in 4 helices (two on front arrowed), with 430 Å repeat and 143 Å between crowns. (**f**) Fitting of myosin head atomic structure into J-motif. Two heads fit precisely into the motif in a configuration essentially identical to the interacting heads in (**d**). The filament also reveals the position of S2 (unknown in (**d**)), showing that the heads are folded back onto the tail, forming an interacting-heads motif (IHM) (Alamo et al. 2008; Woodhead et al. 2005). (**g**) IHM viewed from reverse side, showing S2 interaction with the blocked head (BH). (**c**) modified from (Dutta et al. 2023); (**e–g**) modified from (Woodhead et al. 2005)

The thick filaments of muscle are polymers of the motor protein myosin 2, a hexamer of two identical heavy chains and two pairs of light chains (Fig. 1a), together with associated regulatory and structural proteins. They function, together with the thin, actin-containing filaments of the sarcomere, to generate force and shortening through cyclic interaction of myosin heads with actin, powered by the hydrolysis of ATP (Geeves and Holmes 1999).

The structure of the vertebrate thick filament was first elucidated by negative staining electron microscopy (EM) (Huxley 1963). In the model proposed based on these data, the elongated, α-helical tails of the myosin molecules (Fig. 1a) lie in the filament backbone, with the motors (myosin heads) on the surface (Fig. 1b), close to neighboring actin filaments. The filaments are bipolar, with antiparallel tail interactions at the filament center and parallel interactions in the two halves. Myosin heads occur along the length of the filament except for a central ‘bare zone’ where polarity reverses, and only tails are present. X-ray diffraction of intact relaxed vertebrate muscle provides further insights (Huxley and Brown 1967), revealing that the myosin heads are quasi-helically organized, with a 430 Å repeat and 143 Å between layers (‘crowns’) of heads (Fig. 1c). Thus, each repeat contains 3 crowns (3 × 143 = 430 Å). This ordered structure contrasts with a disordered appearance of heads in the negative stain images, probably due to the low temperature of some of the filament preparations (Huxley 1963), causing disorder (Malinchik et al. 1997; Xu et al. 1999), or to disruption of the labile array of myosin heads by the carbon substrate used to support the sample for negative staining (Craig 2012; Knight and Trinick 1984; Nguyen et al. 2025; Trinick and Elliott 1982).

Two other proteins are also components of vertebrate thick filaments: myosin-binding protein C (MyBP-C) (Heling et al. 2020; Offer et al. 1973) and titin (Gregorio et al. 1999) (Fig. 1c). Both consist of linear strings of fibronectin type 3-like (Fn) and immunoglobulin-like (Ig) domains. MyBP-C, containing 10 (skeletal) or 11 (cardiac) domains is confined to, and defines, the C-zone of the thick filament**,** where it occurs at every third level of heads, matching their 430 Å repeat (Fig. 1c); it is absent from the proximal (P-) zone, adjacent to the bare zone, and from the distal (D-) zone, at the filament ends (Squire 1981). MyBP-C modulates contractile activity in cardiac muscle (Heling et al. 2020). Titin, a giant protein extending from the M-line along the thick filament then through the I-band to the Z-line (Fig. 1c), is organized in an 11-domain super-repeat of Fn and Ig domains in the C-zone, again matching the 430 Å repeat of myosin and MyBP-C (Gregorio et al. 1999). It serves as a template and molecular ruler for thick filament assembly (Bennett et al. 2020; Tonino et al. 2017).

The details of myosin head conformation, the organization of myosin tails in the filament backbone, and the arrangement of MyBP-C and titin were not revealed in these early studies. X-ray diffraction patterns of intact muscle were modeled to elucidate the likely arrangement of the different components (e.g. Harford and Squire 1986; Malinchik and Lednev 1992; for recent update see (Koubassova et al. 2025)), and theoretical models of thick filament structure were proposed (Pepe 1967; Squire 1973; Wray 1979). Parallel progress came from improved negative staining techniques and using more stable invertebrate thick filaments (Craig 2012; Crowther et al. 1985; Kensler and Levine 1982; Vibert and Craig 1983) as well as improvements in vertebrate filament observations (Stewart and Kensler 1986). These EM studies were the first to directly reveal helically ordered arrays of myosin heads, but were limited to ~ 50 Å resolution, and did not disclose any information on tail, titin or MyBP-C organization in the filament.

Two major breakthroughs in understanding thick filament structure came from cryo-electron microscopy (cryo-EM). The first was from a study of purified myosin molecules, the second from native thick filaments. Two-dimensional crystals of purified smooth muscle myosin and heavy meromyosin (HMM), a proteolytic fragment of myosin lacking the distal 2/3 of the tail, showed that myosin’s two heads interacted with each other, asymmetrically, when in the switched-off state (Fig. 1d) (Liu et al. 2003; Wendt et al. 2001), a finding later confirmed and expanded by negative staining of single myosin molecules (Burgess et al. 2007). These asymmetric interactions appeared to inhibit activity of the two heads, in different ways (Wendt et al. 2001). In the ‘blocked’ head (BH), interaction with actin was blocked through binding of its actin-binding region to the ‘free’ head (FH). The actin-binding site was open on the free head, but its ATPase activity appeared to be inhibited by binding to the blocked head.

Despite these intriguing findings, it was questioned whether the head-head interactions observed in crystals were present in native myosin molecules (Sheng et al. 2003). This question was definitively answered by cryo-EM of native tarantula thick filaments, chosen for the superior stability of their myosin head arrays, which clearly showed head-head interactions in 3D reconstructions, exactly like those in the 2D crystals (Woodhead et al. 2005) (Fig. 1e, f). The 3D reconstruction showed, in addition, that the interacting heads were bent back onto the myosin tail (not visible in the 2D crystals), the blocked head interacting with its proximal region (subfragment 2, S2) (Fig. 1f, g). This ‘interacting-heads motif’ (IHM; (Alamo et al. 2008)) appeared to correlate with, and possibly underlie, the ‘super-relaxed’ (SRX) state of muscle (Cooke 2011; Craig and Padron 2022; Stewart et al. 2010), in which ATP turnover by myosin is highly inhibited, conserving energy.

These studies were carried out before the cryo-EM ‘resolution revolution’ (Kuhlbrandt 2014) and were limited to detail at the 20 Å level, leaving many unknowns about the precise interactions of the heads and the detailed organization of the tails. The question also remained: is the IHM present in vertebrate as well as invertebrate muscle? This was answered initially by low resolution (30–40 Å) negative stain observations of mouse (Zoghbi et al. 2008) and human (Al-Khayat et al. 2013) cardiac thick filaments, which showed clear evidence for the IHM and signs of the organization of titin and MyBP-C. The IHM has since been demonstrated in every thick filament (vertebrate and invertebrate (Alamo et al. 2017a)) and every myosin 2 molecule (skeletal, cardiac, smooth and nonmuscle) that has been studied (Jung et al. 2008a, b; Lee et al. 2018).

**Fig. 2:**
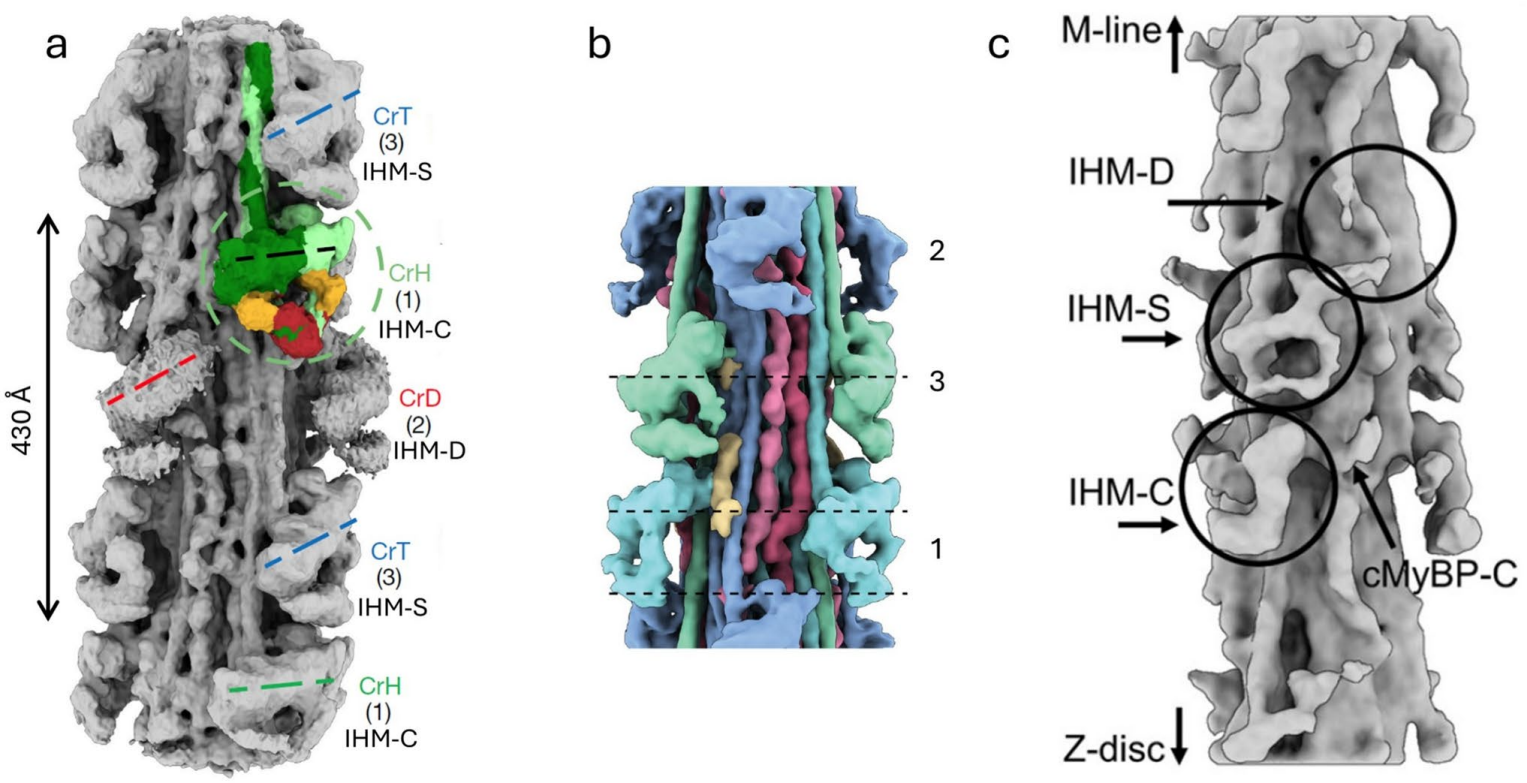
Averaged 3D maps of C-zone structure from the 3 cryo-EM studies. (**a**) Reconstruction of 5 crowns of IHMs in human cardiac thick filament, based on single particle analysis of isolated filaments in presence of mavacamten (Dutta et al. 2023). The structure repeats every 3 crowns (430 Å). Using the naming scheme of (Dutta et al. 2023), Crown T (CrT) has *tilted* IHMs, crown H (CrH) has *horizontal* IHMs, crown D (CrD) has (partially) *disordered* IHMs (wispy surface). The labels below CrT, CrH and CrD show the naming schemes for the equivalent crowns from the reconstructions in (Tamborrini et al. 2023) (**b**) and (Chen et al. 2024) (**c**), respectively. (**b**) Reconstruction of 430 Å repeat of C-zone (3 crowns) of mouse cardiac thick filament based on cryo-ET of relaxed myofibrils in presence of mavacamten (Tamborrini et al. 2023). Crowns 1, 2 and 3 correspond to crowns CrH, CrD and CrT (see (**a**)). Titin is pink, MyBP-C is yellow. (**c**) Reconstruction of 5 crowns of human cardiac thick filament based on cryo-ET of frozen human ventricle fragments in absence of mavacamten (Chen et al. 2024). Crowns IHM-D, IHM-S and IHM-C correspond to crowns CrD, CrT and CrH in (**a**). Reconstructions shown to scale and all with M-line/bare zone towards top; for direct comparison of the three aligned structures, see Fig. S1. (**a**) modified from (Dutta et al. 2023); (**b**) from (Tamborrini et al. 2023); (**c**) From (Chen et al. 2024), reproduced under Creative Commons license https://creativecommons.org/licenses/by-nc-nd/4.0/)

Our knowledge of vertebrate thick filament molecular structure remained at this level until the past two years, when three cryo-EM structures of vertebrate cardiac thick filaments were published, using the most recent microscope and detector hardware and advanced image processing and computer capabilities (Table 1) (Chen et al. 2024; Dutta et al. 2023; Tamborrini et al. 2023). These structures, as high as 6 Å in resolution, reveal exquisite detail of myosin IHMs, myosin tails, and the organization of titin and MyBP-C (Fig. 2). While they agree on most of the key structural features, they differ in some details. Below, we review their major similarities and the likely reasons for their differences.

**Table 1:** Comparing the 3 thick filament cryo-EM structures

	Dutta	Tamborrini	Chen
Technique	Single particle cryo-EM	Cryo-ET	Cryo-ET
Species	Human	Mouse	Human
Preparation	Isolated filament – no actin	In situ – actin present	In situ – actin present
Regions studied	C-zone	C-zone, P-zone, bare zone	C-zone
Resolution	5–6 Å	18–24 Å	16–26 Å
Drug	Mavacamten	Mavacamten	None
Specimen temperature	25°C	25°C	4°C
Ordering of IHMs	CrH, CrT ordered; CrD partially disordered	All IHMs ordered	Most IHMs disordered CrD *most* disordered
MyBP-C orientation	Longitudinal	N-terminal projects to actin at C7	N-terminal projects to actin at C7
Paths of tails associated with different crowns	3 paths – core (CrT), surface (CrD), intermediate (CrH)	Same 3 tail paths	Same 3 tail paths

### The three structures

In two papers published back-to-back, thick filaments were either isolated under relaxing conditions from human cardiac ventricular muscle and studied by single particle cryo-EM (Dutta et al. 2023), or observed in situ in the filament lattice of intact relaxed myofibrils from mouse cardiac muscle by cryo-electron tomography (cryo-ET) (Tamborrini et al. 2023). Both studies used the myosin inhibitor mavacamten to stabilize the labile myosin head array (Anderson et al. 2018). In a subsequent paper, cryo-ET was used to observe thick filament structure in tissue fragments from human cardiac muscle, in the absence of any drug (Chen et al. 2024). The resolutions of the structures were ~ 6 Å, 18 Å and 26 Å, respectively. The better resolution of the isolated filaments (Dutta et al. 2023) reflects the larger number of particles that were averaged compared with the cryo-ET observations (Chen et al. 2024; Tamborrini et al. 2023). All studies focused on the C-zone of the filament (Fig. 1c), where myosin heads are best ordered, while one revealed, in addition, the structure of the P-zone, M-line and bare-zone (Tamborrini et al. 2023). None provided information on the D-zone, where myosin heads are thought to be poorly ordered (Brunello et al. 2020). Here, we compare the C-zone structures from the three studies, available as cryo-EM maps (see Figure legends) and in two cases as atomic models (PDB 8g4l (Dutta et al. 2023), PDB 8q6t (Tamborrini et al. 2023)).

### Similarities between the structures

Overall, there was excellent agreement between the three structures. This was especially striking for the two initial studies (Dutta et al. 2023; Tamborrini et al. 2023), which were conducted completely independently, giving confidence in the major structural features revealed and their interpretation.

#### Arrangement of IHMs

All three threefold symmetric structures showed myosin heads in the IHM conformation (Figs. 2, S1). In the first two (Dutta et al. 2023; Tamborrini et al. 2023), essentially all heads formed IHMs, well-ordered in quasi-helices. In the third (Chen et al. 2024), ~ 90% of heads were disordered, but IHMs were still seen (~ 10% of heads), sometimes as “semi-IHMs”, with only one head visible. In all three studies, each of the three crowns in the 430 Å repeat of the C-zone had a different IHM orientation and interactions and, in two of the studies (Chen et al. 2024; Dutta et al. 2023), different stabilities. These suggest different functional capabilities in the 3 crowns. Using the terminology of (Dutta et al. 2023) (Figs. 2, S1), crown T (CrT) exhibited IHMs that were *Tilted* (the interacting motor domains formed a density that was at an angle to the filament axis). In crown H (CrH), the line joining the motor domains was approximately *Horizontal* (viewed with the filament oriented vertically). In crown D (CrD), IHMs were also tilted, but were weaker in density and exhibited a noisy surface in the isolated filament study (Dutta et al. 2023); these were interpreted to be (partially) dynamically *Disordered*. The third study (Chen et al. 2024) reported almost complete disorder in CrD, while CrD was considered to be ordered in (Tamborrini et al. 2023) (see later for explanation). The reason for the greater order of CrT and CrH is likely to be interactions of the FHs with MyBP-C in these two crowns that are absent from CrD. The crowns corresponding to CrH, CrD, CrT (Dutta et al. 2023) were given different names in the other two studies: respectively Crown 1, Crown 2, Crown 3 (Tamborrini et al. 2023), and IHM-C, IHM-D, IHM-S (Chen et al. 2024) (Fig. 2); for the remainder of this review we will use CrT, CrH and CrD (Tilted, Horizontal, Disordered) (Dutta et al. 2023).

#### Arrangement of tails

The 20-Å diameter myosin tails were clearly distinguished and could be followed for their entire lengths in all three structures. In the single particle reconstruction (Dutta et al. 2023), the individual α-helices of each tail were well-resolved, such that local structural features (e.g. skip residues in the sequence) could be discerned. At the lower resolution of the cryo-ET studies (Chen et al. 2024; Tamborrini et al. 2023), the tails could be followed but their internal structure was not revealed. The tails in all 3 structures ran longitudinally, with a slight twist around the filament axis. The 3 studies showed full agreement on the three strikingly different courses followed by the tails associated with the 3 types of IHM. Tails originating from CrT IHMs started at the surface and traveled to the center of the filament, where they formed the filament core. Tails of CrH IHMs dipped towards the center, then rose again to the surface. Tails from the CrD IHMs remained at the surface for their entire length, where they formed a docking platform for MyBP-C. Surface charge analysis based on the atomic model (Dutta et al. 2023) suggested that neighboring tails staggered by odd multiples of 143 Å interacted with each other electrostatically as predicted 40 years earlier based on sequence analysis (McLachlan and Karn 1982).

#### Arrangement of MyBP-C

In the isolated thick filaments (Dutta et al. 2023) and mouse myofibrils (Tamborrini et al. 2023), MyBP-C was identified by flexible fitting of AlphaFold structures (Jumper et al. 2021) of individual MyBP-C domains to a 430-Å repeating density observed on the surface of the filament; in the third study, Chen et al. interpreted their map by fitting the C-zone atomic model 8g4l (Dutta et al. 2023) to their data (Chen et al. 2024). These fittings revealed that the C-terminal domains of MyBP-C (domains C10-C8) ran longitudinally along the filament surface, attached to the thick filament via a docking site formed by tails from three adjacent levels of CrD IHMs staggered by 430 Å (Dutta et al. 2023; Tamborrini et al. 2023).

**Fig. 3:**
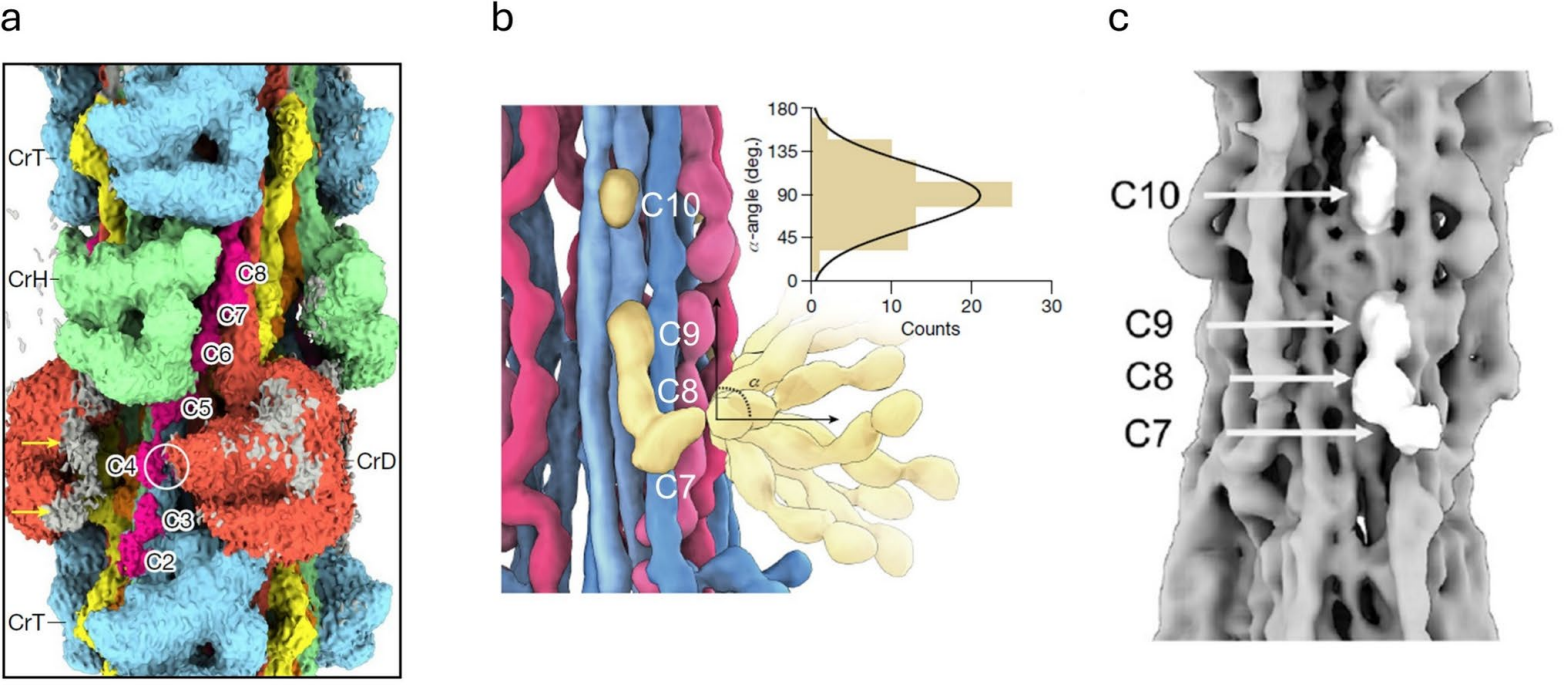
3D reconstructions of the C-zone from the 3 studies showing organization of MyBP-C domains. (**a**) In isolated thick filament (Dutta et al. 2023), MyBP-C (pink) runs longitudinally along the surface of the filament from C10-C2. C10 and C9 are hidden behind the CrT IHM. Titin strands orange, yellow. (**b**) In intact myofibril (Tamborrini et al. 2023), with thin filaments surrounding thick filaments, the C-terminal domains C10-C8 run longitudinally, while the molecule pivots at C7 to extend transversely, at varying angles, towards the thin filament. MyBP-C yellow, tails blue, titin pink. (**c**) In intact myofibril in absence of mavacamten (Chen et al. 2024), the organization of MyBP-C domains is similar to that in its presence (**b**): C10-C8 run longitudinally, while at C7 the molecule pivots to run transversely. (**a**) from (Dutta et al. 2023); (**b**) modified from (Tamborrini et al. 2023); (**c**) from (Chen et al. 2024), reproduced under Creative Commons license https://creativecommons.org/licenses/by-nc-nd/4.0/)

Strikingly, no interaction of MyBP-C with titin was seen in any of the reconstructions. This contrasts with an expected interaction of MyBP-C’s C10 domain with the T1 domain of titin, based on in vitro observations (Freiburg and Gautel 1996). Thus, titin positions MyBP-C *indirectly*, by determining the positions of the CrD tails that form the docking site. A key difference between the reconstructions was the orientation of the region of MyBP-C N-terminal to the C8 domain. In the isolated filament (single particle) reconstruction (Dutta et al. 2023), domains C7-C5 ran along the backbone, continuing the longitudinal orientation of C10-C8 (Fig. 3a), with C6 also binding to the docking site on CrD tails. Weaker density, extending as far as the C2 domain (Fig. 3a), suggested that domains C4-C2 also run longitudinally, but are more mobile/less strongly bound to the thick filament surface. Contrasting with this longitudinal orientation, the cryo-ET studies (Chen et al. 2024; Tamborrini et al. 2023), in which thick filaments are present within the native filament lattice, showed the more N-terminal region of MyBP-C, starting at C7, bending radially away from the thick filament, hinging at the linker between C8 and C7, and projecting towards neighboring actin filaments (Fig. 3b, c; see below).

#### Arrangement of titin

In all three reconstructions, pairs of kinked and slightly curved titin strands ran parallel to each other in each sector of the threefold symmetric filament (Fig. 2) (Chen et al. 2024; Dutta et al. 2023; Tamborrini et al. 2023). As with MyBP-C, individual titin domains in the 11-domain, 430 Å super-repeat (Gregorio et al. 1999) were identified by fitting AlphaFold domain structures to the map densities (Dutta et al. 2023; Tamborrini et al. 2023) or by fitting titin from the atomic model 8g4l (Dutta et al. 2023) to the titin strands (Chen et al. 2024); all three studies agreed on the identification of the specific domains. Both titin strands showed numerous interactions with myosin tails. Most of the interactions were with CrH and CrD tails, with few interactions with CrT tails. Surface charge analysis (Dutta et al. 2023) suggested that titin-tail interactions were electrostatic, the ~ 42-Å repeat of titin domains matching the ~ 42-Å charge repeat along the myosin tails (McLachlan and Karn 1982). Thus, the 430-Å super-repeat of 11 titin domains in the C-zone appears to position the myosin tails in their unique locations, supporting titin’s role as a thick filament template (Gregorio et al. 1999; Tonino et al. 2017). The electrostatic interactions between neighboring tails staggered by odd multiples of 143 Å (Dutta et al. 2023; McLachlan and Karn 1982) appear to reinforce this titin-based arrangement.

### Explaining differences between the structures

Apart from resolution, the main differences between the structures are the orientation of the N-terminal half of MyBP-C and the degree of ordering of the IHMs. These are likely due to the different environments of the thick filaments (isolated or within the myofibrillar lattice) and the different methods of specimen preparation. Another difference is the use of mouse (Tamborrini et al. 2023) *vs.* human cardiac muscle (Chen et al. 2024; Dutta et al. 2023). In this regard it is striking that, despite their tenfold difference in heart rate, both species have thick filaments with essentially the same atomic structure.

#### Orientation of MyBP-C

The most striking difference is the organization of MyBP-C. The longitudinal orientation observed with single particle cryo-EM of isolated filaments (Dutta et al. 2023), compared with the radially projecting orientation of the N-terminal domains in cryo-ET of myofibrils (Chen et al. 2024; Tamborrini et al. 2023) (Fig. 3) is not a discrepancy, but likely reflects the difference in thick filament environments between techniques. When thin filaments overlap thick filaments, as in myofibrils (cryo-ET), actin binding of MyBP-C’s N-terminus is possible (Heling et al. 2020; Huang et al. 2023; Luther et al. 2011), stabilizing the radially extended conformation; when thick filaments are isolated from thin filaments (single particle cryo-EM), actin is absent and N-terminal binding is not possible.

There is much past evidence for MyBP-C binding to actin (Luther et al. 2011; Rahmanseresht et al. 2021; Risi et al. 2022; van Dijk et al. 2014), modulating thick-thin filament interaction in muscle (Heling et al. 2020). Extension of MyBP-C’s N-terminal towards actin in the cryo-ET studies (Fig. 3b, c) (Chen et al. 2024; Tamborrini et al. 2023) dramatically demonstrates such interaction in resting muscle in situ. In contrast, the isolated thick filament reconstruction, where thin filaments have been removed (Dutta et al. 2023), may simulate the H-zone of a stretched sarcomere, where actin filaments have been withdrawn. Without its actin binding partner, the MyBP-C N-terminus may become disordered (Reconditi et al. 2014) or bind to the thick filament. The latter was observed for domains C6-C2 in the isolated filament reconstruction (Fig. 3a) (Dutta et al. 2023) and is consistent with in vitro studies of N-terminal interaction with myosin heads or S2 (Heling et al. 2020; Nag et al. 2017; Ratti et al. 2011). Cardiac MyBP-C is typically phosphorylated, in the M-domain (near the N-terminus) in normal muscle, which can affect both its myosin and actin affinity (Heling et al. 2020). Phosphorylation levels were not determined in the three studies, and possible differences may contribute to the differing orientations of MyBP-C between the isolated filament and sarcomeric structures. Importantly, these contrasting orientations support models of MyBP-C function in which the N-terminal domains can switch binding partners depending on proximity/absence of actin and/or phosphorylation of the M-domain (Heling et al. 2020).

#### Effect of mavacamten on IHM ordering

Another key difference between the reconstructions is the degree of ordering of IHMs with and without mavacamten. In the presence of mavacamten, most heads are ordered, in both myofibrils (Tamborrini et al. 2023) and isolated filaments (Dutta et al. 2023) (Figs. 2a, b, S1a, b). A dramatically different appearance is seen without mavacamten, where ~ 90% of heads are disordered, and only a small fraction show complete or semi-IHMs (Chen et al. 2024) (Fig. S2). Does this imply that mavacamten pushes myosin heads into ordered IHMs and that relaxed cardiac muscle in the absence of drug has mostly disordered heads? Available evidence argues against this view. The mavacamten-free specimens used for cryo-ET were prepared at low ambient temperature (4 °C; Liang Chen, personal communication). Low temperature is known from X-ray diffraction to cause a high degree of disordering of myosin heads from the quasi-helical positions that they occupy at near physiological temperature (Malinchik et al. 1997; Morotti et al. 2024; Ovejero et al. 2022; Xu et al. 1999). It therefore seems likely that low temperature, not the absence of mavacamten, caused the disordering; in fact, these cryo-ET observations provide a striking visual demonstration of disorder that had previously only been inferred from X-ray data.

X-ray studies of cardiac muscle in the presence and absence of mavacamten support this conclusion (Anderson et al. 2018; Koubassova et al. 2025). At near-physiological temperature (> 20 °C), the intensities of myosin layer lines M1–M6, a measure of helical ordering, were reduced by only 14–29% in the absence of mavacamten compared to those in its presence; the strongest layer line (M1, corresponding to the 430 Å repeat) was reduced by only 13% (27% reported previously (Anderson et al. 2018)). This reflects only a 7% (Koubassova et al. 2025)−15% (Anderson et al. 2018) reduction in amplitude in the absence of mavacamten, suggesting at most a mild stabilization of helical order in its presence (Koubassova et al. 2025). The distribution of intensity along the layer lines was also the same (Anderson et al. 2018; Koubassova et al. 2025), implying no major change in IHM structure. These figures compare with the much greater (90%) loss of intensity when cardiac muscle is cooled from ~ 35 to < 10 °C (Morotti et al. 2024; Ovejero et al. 2022), reflecting a near total loss of helical order—consistent with the low temperature cryo-ET findings (Chen et al. 2024). We conclude that mavacamten does not significantly alter IHM structure and is not required for IHM formation or helical ordering. The ordered, IHM-based structure in the presence of mavacamten (Dutta et al. 2023; Tamborrini et al. 2023) is therefore likely to be close to the physiologically relaxed structure of the cardiac thick filament.

Multiple other studies at near-physiological temperature support the ordering of heads in the IHM conformation in the absence of mavacamten. Three-dimensional reconstruction of negatively stained human cardiac thick filaments, without any drug, shows myosin heads in quasi-helically ordered IHMs in the C-zone (Al-Khayat et al. 2013). Cryo-EM of cardiac myosin constructs, consisting of the two heads and a short tail, shows IHMs in the absence as well as the presence of mavacamten, with only subtle differences between the two (Grinzato et al. 2023; McMillan et al. 2025; Somavarapu et al. 2025). Isolated IHMs in the absence of mavacamten (Grinzato et al. 2023; Somavarapu et al. 2025) closely match the CrH and CrT IHMs in the thick filament in its presence (Dutta et al. 2023), suggesting a minimal effect of mavacamten on IHM structure. Fluorescence polarization studies imply ordering of myosin heads in the IHM in skinned, relaxed muscle fibers in the absence of any drug (Fusi et al. 2015).

#### Stability and location of CrD heads

Another difference between the reconstructions is the degree of order and the precise location specifically of the CrD IHMs. This difference is subtle, but may have crucial functional consequences. In the isolated filament (Dutta et al. 2023), crown CrD of the 430-Å triplet repeat has lower density and exhibits a noisier surface than crowns CrT and CrH (Figs. 2a, S1). This suggests that CrD is partially disordered, although on average it retains a similar IHM conformation to the two stable crowns. In contrast, cryo-ET of myofibrils in the presence of mavacamten is stated to show little difference in ordering of CrD compared with CrT and CrH (Tamborrini et al. 2023). How can these results be reconciled? The cryo-ET study (Tamborrini et al. 2023) shows that CrD IHMs in the two proximal 430-Å repeats of the C-zone (crowns A3-A8 of (Tamborrini et al. 2023)) are different from those in the main region of the C-zone. It was therefore suggested (Tamborrini et al. 2023) that the weaker, noisier CrD IHMs in the isolated filament (Dutta et al. 2023) might arise from averaging such different IHMs, not from disorder of CrD IHMs themselves.

We have tested this possibility (Dutta et al., in preparation) by separating the particles used in (Dutta et al. 2023) into ten 3D subclasses (Fig. S3a). Most of the classes, including the majority Class 5, show a weak and disordered CrD and appear similar to the averaged reconstruction obtained using all the particles (Fig. 2a). A small subset (Class 7 and possibly Class 1), differing from the others but similar to each other, show a stronger CrD together with IHM and MyBP-C features and interactions similar to those observed by cryo-ET of repeat 2 of (Tamborrini et al. 2023). The particles creating these two classes are excluded from Class 5, which still exhibits a noisy surface and lower density for CrD (Fig. S3a, class 5), similar to the original global average (Fig. 2a). Thus, the noise and lower density do not appear to arise from averaging different structures, but are real.

It remains possible that the difference in stability of CrD in isolated and sarcomeric thick filaments is real, and that we should not assume that the structure is the same in both environments: myosin heads in the filament lattice, closely surrounded by thin filaments and other thick filaments, may be more stable than those in isolated filaments. However, the sarcomeric observations in the absence of mavacamten (Chen et al. 2024) argue against this. While low temperature is the likely cause of global disordering of the heads in that study (discussed above), there is a dramatic difference in the degree of disordering between the different crowns (Fig. S2): crowns CrT and CrH show full IHMs in 10% of 3D classes, while CrD crowns show no full IHMs, and only 5% that have any order at all, as semi-IHMs—strong evidence for lability of CrD, even in the myofibrillar lattice. Modeling of the X-ray diffraction pattern of skeletal and cardiac muscles adds further support, showing that the best match of different thick filament models to the observed X-ray patterns of intact muscle occurs when CrD is partially disordered (Koubassova et al. 2025, 2022).

If this is the case, how do we explain the absence of the noise features in the cryo-ET study (Tamborrini et al. 2023), when they are obvious in the single particle reconstruction (Dutta et al. 2023)? The difference may be a function of resolution: when the 6 Å resolution single particle reconstruction is filtered to the 18 Å resolution of the cryo-ET study, these fine features disappear (Fig. S3c), and CrD looks similar to the averaged crown 2 found by cryo-ET (Fig. S3b) (Tamborrini et al. 2023), with weaker density by both methods, further supporting a degree of disordering of CrD (Fig. S3b, c).

**Fig. 4:**
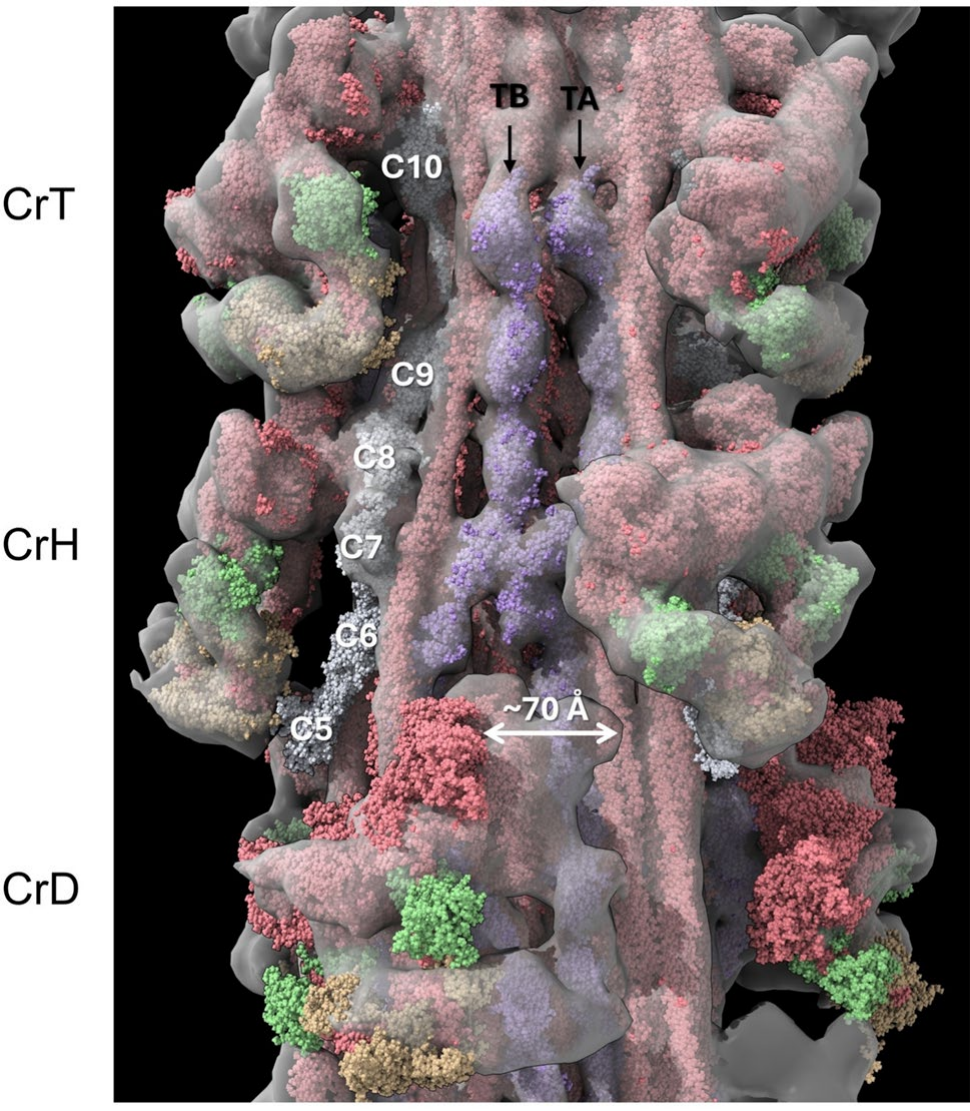
CrD position is different in isolated filament compared with filament in myofibrillar lattice. The atomic model from the isolated filament reconstruction PDB 8g4l (Dutta et al. 2023) is fitted into the translucent cryo-ET map of the C-zone of the thick filament in the filament lattice (EMD-18146; (Tamborrini et al. 2023)). The fit demonstrates the excellent agreement between the two structures in the positions of the CrH and CrT IHMs, the tails (red), titin (TA and TB, purple), and of MyBP-C (grey) from domain C10 to C8, after which the two diverge. C7 projects away from the filament in the cryo-ET map while domains C7-C5 continue along the surface of the isolated filament reconstruction. Viewing in three dimensions is required to fully appreciate this difference (Movie S1). The structures are strikingly different for CrD, which is displaced ~ 70 Å azimuthally between the two (double arrowhead). This suggests a substantial influence of neighboring thin filaments on CrD position

The putative disorder of CrD is important because of its potential role in muscle activation. We suggest that the disorder is dynamic and may relate to the weakness or absence of CrD interactions with MyBP-C (Dutta et al. 2023) compared with the stabilizing interactions seen with CrT and CrH that are thought to promote the SRX state in the C-zone (Dutta et al. 2023; McNamara et al. 2016; Nelson et al. 2023). In addition to their weaker density and noisier surface, motion of CrD IHMs is also suggested by a flattening of their S2 density not observed with CrT or CrH S2 (Dutta et al. 2023). CrD heads, loosely bound to the thick filament surface, may serve as sentinels that interact transiently with actin filaments to detect their activation state (Kalakoutis et al. 2025) by searching for myosin binding sites exposed by Ca^2+^-activated tropomyosin movement (Fusi et al. 2015; Woodhead and Craig 2015). This concept is supported by the elevated position of CrD IHMs above the backbone surface, through binding to a raised region of titin B (Dutta et al. 2023). Further, dramatic support for this concept comes from a striking difference in the position of CrD in thick filaments surrounded by thin filaments in the cryo-ET study of the myofibrillar lattice (Tamborrini et al. 2023) compared with filaments isolated from the lattice (Dutta et al. 2023). Fitting the isolated filament atomic model, PDB 8g4l, into the C-zone density map from cryo-ET reveals a precise match of CrH, CrT, tails, titin, and the C-terminal domains of MyBP-C, as discussed earlier. However, CrD is displaced ~ 70 Å between the two structures (Fig. 4), implying a direct influence of actin on CrD position, consistent with CrD’s putative sentinel role in muscle activation. Disordered, loosely-bound heads in the D-zone (lacking MyBP-C) may also serve a sentinel role (Irving 2025).

### Implications

The three cryo-EM studies of the cardiac thick filament reviewed here are strikingly complementary. Analysis of isolated filaments (Dutta et al. 2023) provides the highest resolution – but of a filament removed from the sarcomere – while cryo-ET shows filaments, at lower resolution, in their native surroundings and reveals differences in structure along the thick filament (Chen et al. 2024; Tamborrini et al. 2023). While mavacamten helped to provide the most detailed information (Dutta et al. 2023; Tamborrini et al. 2023), the structure in its absence provides additional insights (Chen et al. 2024), including the existence of “semi-IHMs”, in which one head is ordered and the other disordered; in the case of CrH, this directly demonstrates the predicted (Dutta et al. 2023; Tamborrini et al. 2023) stabilizing effect of MyBP-C on the FH. A third cryo-ET study showed filaments in the native environment of a live cardiomyocyte (Woldeyes et al. 2025), but details of thick filament structure were lacking, possibly due to active beating of the cells. These cryo-EM studies are complemented by other biophysical techniques. X-ray diffraction of intact muscle provides a signal from the thick filaments in situ (Ma and Irving 2022) but requires EM information for interpretation: the C-zone atomic model PDB 8g4l, based on cryo-EM (Dutta et al. 2023), quantitatively explains the X-ray pattern of cardiac muscle (Koubassova et al. 2025), thus supporting the presence of thick filaments in relaxed muscle in situ having the cryo-EM structure seen in Fig. 2a. X-ray crystallography yields the high-resolution structure of isolated myosin heads (Dominguez et al. 1998; Houdusse et al. 2000; Rayment et al. 1993), but not the 2-headed IHM. This gap is filled by cryo-EM of 2-headed myosin constructs, which show the isolated IHM at high resolution (Grinzato et al. 2023; McMillan et al. 2025; Somavarapu et al. 2025). Fluorescence polarization provides a signal from intact, relaxed, skinned muscle (Fusi et al. 2015), that supports the organization of IHMs seen in the cryo-EM studies (Dutta et al. 2023; Tamborrini et al. 2023). And single ATP-turnover studies of the SRX state in filaments in situ (Cooke 2011) and in myosin constructs in solution (Anderson et al. 2018) correlate well with direct observation of the IHM by EM (Craig and Padron 2022; Nguyen et al. 2025; Rasicci et al. 2022).

The new thick filament structures provide critical new insights into cardiac muscle function and potential disease mechanisms. Past in vitro work suggested that the N-terminal region of MyBP-C was the “functional” part of the molecule (with sites for phosphorylation and for interaction with actin and myosin heads), while the C-terminal functioned simply to anchor the molecule to the thick filament (Heling et al. 2020). The cryo-reconstructions suggest that the C-terminal plays a much more active role, through its interactions with CrH and CrT IHMs (Dutta et al. 2023; Tamborrini et al. 2023), which regulate their activity in the native filament and could account for their enhanced stability and the stronger SRX state in the C-zone (McNamara et al. 2016; Nelson et al. 2023). The complex interactions of MyBP-C, myosin, and titin observed in the reconstructions suggest a possible mechanism for the basic property of length-dependent activation in cardiac muscle (Starling’s law (de Tombe et al. 2010; Dutta et al. 2023)): the kinked structure of titin straightens when the sarcomere is extended, disrupting MyBP-C’s stabilizing connections to IHMs and releasing more heads for force production.

The cryo-EM based atomic model also provides novel insights into the structural basis of cardiomyopathies caused by variants in the myosin heavy chain and MyBP-C (genes *MYH7* and *MYBPC3*, respectively) (Dutta et al. 2025, 2023). Pathogenic variants in these proteins that cause hypertrophic cardiomyopathy (HCM), when mapped onto the model (PDB 8g4l), are significantly clustered at interfaces between thick filament components, including head-head and head-S2 interfaces of the IHM and interfaces involving myosin tails, titin, and MyBP-C (Dutta et al. 2025). Disruption of these interfaces by mutations would likely destabilize IHMs and release heads for interaction with actin, explaining the impaired relaxation that characterizes HCM (Nag et al. 2017; Sarkar et al. 2020; Spudich et al. 2024). Some dilated cardiomyopathy (DCM) mutations also occur at interfaces, but in this case strengthen the IHM, explaining impaired contraction in this phenotype (Rasicci et al. 2022; Sharma et al. 2025). These analyses extend the mapping of mutations on the head-head and head-S2 interfaces in the isolated IHM (Alamo et al. 2017b; Grinzato et al. 2023) to now include all thick filament components in their native context.

### Future studies

The studies reviewed here open the way to further insights into thick filament structure. **(1)** X-ray diffraction patterns of skeletal and cardiac muscle are similar, suggesting comparable filament structures (Huxley and Brown 1967; Ma et al. 2018; Ma and Irving 2022; Matsubara 1980). Cryo-EM of skeletal filaments will test this idea and reveal any differences specific to the skeletal system. **(2)** We know little about the bare zone and the distal (D-) zone of thick filaments (Squire 1981). While the bare zone (the central, headless region of polarity reversal) was analyzed in (Tamborrini et al. 2023), a high-resolution structure is not available, but should be possible by single particle cryo-EM of isolated filaments. Heads in the 300-nm-long D-zone at each end of the filament, lacking MyBP-C, are less ordered than in the C-zone (Brunello et al. 2020), but may still be amenable to cryo-EM or cryo-ET reconstruction. **(3)** We currently lack a high-resolution structure of the C-zone in the absence of drug and at near-physiological temperature. Past, low-resolution studies show that myosin heads are ordered without drug (Al-Khayat et al. 2013; Kensler et al. 2017). Single particle cryo-EM studies in the absence of mavacamten may therefore yield resolution similar to that in its presence. **(4)** In addition to mapping mutations onto the atomic model as discussed above, it should be possible to determine their impact on filament structure experimentally in cases where they produce stable filaments (disordered filaments may produce little detail). Heart tissue from human subjects will normally contain heterozygous mutations, which may be difficult to analyze if mutant protein is randomly mixed with wild type. Homozygous mouse models will facilitate analysis (e.g. (Zoghbi et al. 2008)). **(5)** Finally, myosin-binding protein H (MyBP-H) and MyBP-H-like (MyBP-HL) are expressed along with, or substituting for, MyBP-C in different situations. MyBP-H is expressed in large amounts in zebrafish skeletal muscle (Mead et al. 2024), while MyBP-HL is present in mammalian atria (Barefield et al. 2023). The similarity of these MyBP-C homologs, corresponding to the C-terminal 4 (MyBP-H) and 3 (MyBP-HL) domains of MyBP-C, suggests that they may be nicely visualized, and their function therefore elucidated, in cryo-EM studies of these filaments.

### Dedication

We dedicate this review to the memory of Dr. Roger Cooke, a sorely missed friend, collaborator, and colleague. His seminal discovery and investigation of the SRX state remain central to the muscle field today, most importantly in their connection to the interacting-heads motif (Cooke 2011; Craig and Padron 2022), so beautifully revealed at near-atomic resolution in the thick filament structures unveiled by cryo-EM.

## Supplementary Information

Below is the link to the electronic supplementary material.

ESM1 (DOCX 1.29 MB)

ESM2 (MP4 12.5 MB)

## Data Availability

No datasets were generated or analysed during the current study.
