# Supplementary material for: Comparing cryo-EM structures of the vertebrate cardiac muscle thick filament
Source: Biophys Rev. 2026 Jan 8;18(3):613–25. doi: 10.1007/s12551-025-01393-9 (PMC13089571; doi:10.1007/s12551-025-01393-9)
Supplement: Supplementary file 1 [file 12551_2025_1393_MOESM1_ESM.docx]

**SUPPLEMENTARY MATERIAL**

Comparing cryo-EM structures of the vertebrate cardiac muscle thick filament

Roger Craig, Debabrata Dutta, Natalia A. Koubassova, Andrey K. Tsaturyan, and Raúl Padrón

**Supplementary Figures**

**Fig. S1. Aligned cryo-EM maps of the C-zone of the three thick filament structures in longitudinal (upper) and transverse (lower) views. (a)** EMD-29722 (Dutta et al., 2023); **(b)** EMD-16992 (Tamborrini et al., 2023); **(c)** EMD-40468 (Chen et al., 2024). In longitudinal view, note weaker density and noisy surface of CrD compared with CrT and CrH in **(a)**, weaker density, but no noise features of CrD in **(b)**, and absence of any ordered CrD density in **(c)**—all consistent with dynamic disordering of IHMs in CrD. Compare with **Fig. 2a-c.** Transverse views show more details in backbone of **(a)** compared with **(b)** and **(c)**.

**Fig. S2. IHM organization in absence of mavacamten.** Focused classification and averaging of motifs in cryo-ET reconstruction in absence of mavacamten (Chen et al., 2024). The relevant crown used for the classification is the middle crown of these 430 Å-long segments. The top row is CrH; the next is CrT; the bottom two rows are CrD (IHM-C, IHM-S, and IHM-D of (Chen et al., 2024), respectively). Most heads are disordered at all three levels. Occasional complete IHMs are seen in class 6 of CrH (13% of total) and class 1 of CrT (11% of total); there are no complete IHMs in CrD. Semi-IHMs are seen in class 7 of CrH (8% of total, FH ordered) and class 14 of CrD (5% of total, BH ordered). All other motifs are disordered; the low temperature of specimen preparation is likely to be the cause of the disorder (see text). From (Chen et al., 2024), reproduced under Creative Commons license [https://creativecommons.org/licenses/by-nc-nd/4.0/](https://nam10.safelinks.protection.outlook.com/?url=https%3A%2F%2Fcreativecommons.org%2Flicenses%2Fby-nc-nd%2F4.0%2F&data=05%7C02%7Croger.craig%40umassmed.edu%7Cb45abf3fd0d44973f69d08ddfbaa31c0%7Cee9155fe2da34378a6c44405faf57b2e%7C0%7C0%7C638943430602420292%7CUnknown%7CTWFpbGZsb3d8eyJFbXB0eU1hcGkiOnRydWUsIlYiOiIwLjAuMDAwMCIsIlAiOiJXaW4zMiIsIkFOIjoiTWFpbCIsIldUIjoyfQ%3D%3D%7C0%7C%7C%7C&sdata=g%2BbNDwxuK%2BSDm%2Fz%2BLzVkjpqcKV5UuhDR2kRLQV2AT8c%3D&reserved=0)).

**Fig. S3. CrD is dynamically disordered. (a)** Ten 3D classes obtained from same dataset as in (Dutta et al., 2023) with number of particles shown in thousands (K). Class 5 (the largest, 87K particles) shows CrD as weak and noisy, and CrH and CrT as horizontal and tilted, respectively. Classes 0, 2, 3, 4, 6, 8 and 9 show similar features. In classes 1 and 7 (19K particles total), the upper CrH (yellow circles) appears tilted (not horizontal) and moved to the left azimuthally compared with class 5, while CrD (red circle) is stronger and less noisy. This corresponds to the appearance of the transition region of the C-zone into the P-zone (crowns A5-A7) in (Tamborrini et al., 2023). The large number of particles and high resolution of class 5 suggests that it comes from the regular part of the C-zone defined by Tamborrini (crowns A15-A29); the particles from the transition region, included in classes 1 and 7, are excluded from this class, which appears similar to the reconstruction using all the particles (**Fig. 2a**). Thus, inclusion of particles from the transition zone is not the cause of CrD disorder in the isolated filament reconstruction. This supports the view that CrD is less stable than CrT and CrH. **(b, c)** Comparison of CrD structure in myofibril compared with isolated filament. **(b)** Cryo-ET reconstruction (EMD-16992), resolution ~ 18 Å (Tamborrini et al., 2023). **(c)** Single particle reconstruction (~6 Å resolution) (Dutta et al., 2023) filtered to 18 Å resolution. At this lower resolution, CrD lacks the noisy surface seen at 6 Å resolution (**Figs. 2a, S1a**) and appears similar in shape and weakened density to CrD in the cryo-ET map. These observations are consistent with significant mobility/dynamic disordering of CrD in both reconstructions.

**Supplementary Movies**

**Movie S1. 3D view of fitting of isolated filament atomic model into cryo-ET map of C-zone.** The movie shows the isolated filament atomic model PDB 8g4l (Dutta et al., 2023) fitted into the translucent cryo-ET map of the C-zone of the thick filament in the filament lattice (EMD-18146; (Tamborrini et al., 2023)). Rotation about the filament axis clearly demonstrates the outstanding correspondence of CrT and CrH (top and middle IHMs, respectively), myosin tails, titin and MyBP-C domains C8-C10 in the two structures (see **Fig. 4**). The key differences are the divergence of MyBP-C away from the filament surface starting at C7 in the myofibril, compared with its longitudinal orientation in the isolated filament, and the displacement of CrD by ~70 Å between the two structures.
